# The Impact of Co Doping and Annealing Temperature on the Electrochemical Performance and Structural Characteristics of SnO_2_ Nanoparticulate Photoanodes

**DOI:** 10.3390/ma15196534

**Published:** 2022-09-21

**Authors:** Abeer S. Altowyan, Mohamed Shaban, Khaled Abdelkarem, Adel M. El Sayed

**Affiliations:** 1 Department of Physics, College of Science, Princess Nourah bint Abdulrahman University, P.O. Box 84428, Riyadh 11671, Saudi Arabia; 2 Physics Department, Faculty of Science, Islamic University of Madinah, P.O. Box 170, Al Madinah Al Monawara 42351, Saudi Arabia; 3 Nanophotonics and Applications (NPA) Lab, Department of Physics, Faculty of Science, Beni-Suef University, Beni-Suef 62514, Egypt; 4 Physics Department, Faculty of Science, Fayoum University, El Fayoum 63514, Egypt

**Keywords:** water splitting, spin-coated SnO_2_ films, catalysis, hydrogen generation

## Abstract

Obtaining H_2_ energy from H_2_O using the most abundant solar radiation is an outstanding approach to zero pollution. This work focuses on studying the effect of Co doping and calcination on the structure, morphology, and optical properties of spin-coated SnO_2_ films as well as their photoelectrochemical (PEC) efficiency. The structures and morphologies of the films were investigated by XRD, AFM, and Raman spectra. The results confirmed the preparation of SnO_2_ of the rutile phase, with crystallite sizes in the range of 18.4–29.2 nm. AFM showed the granular structure and smooth surfaces having limited roughness. UV-Vis spectroscopy showed that the absorption spectra depend on the calcination temperature and the Co content, and the films have optical bandgap (*E_g_*) in the range of 3.67–3.93 eV. The prepared samples were applied for the PEC hydrogen generation after optimizing the sample doping ratio, using electrolyte (HCl, Na_2_SO_4_, NaOH), electrode reusability, applied temperature, and monochromatic illumination. Additionally, the electrode stability, thermodynamic parameters, conversion efficiency, number of hydrogen moles, and PEC impedance were evaluated and discussed, while the SnO_2_ films were used as working electrodes and platinum sheet as an auxiliary or counter electrode (2-electrode system) and both were dipped in the electrolyte. The highest photocurrent (21.25 mA/cm^2^), number of hydrogen moles (20.4 mmol/h.cm^2^), incident photon-to-current change efficiency (6.892%@307 nm and +1 V), and the absorbed photon-to-current conversion efficiency (4.61% at ~500 nm and +1 V) were recorded for the 2.5% Co-doped SnO_2_ photoanode that annealed at 673 K.

## 1. Introduction

Developing an efficient and clean energy technology with zero pollution became the centre of attention to meet the energy demand at the time of fossil fuel depletion. As an outstanding approach, converting renewable solar energy to highly efficient, eco-friendly, and affordable H_2_ energy using H_2_O splitting is attracting intensive research interest worldwide [1,2]. In addition to CO_2_ zero-emission, the H_2_ energy value is ~122–142 kJ/g which is 2.8–3.0 times larger than the energy value obtained from liquid hydrocarbons used as fuel [3,4]. In this context, many attempts have been carried out to adapt various transparent metal oxides (TCOs) as photoanode in the electrochemical cell for H_2_ energy generation. Among them is a stannic oxide (SnO_2_).

SnO_2_ is nontoxic, highly transparent, abundant in nature, has good electrical conductivity, and has a high electron mobility of ~(1–2) × 10^2^ cm^2^/V·s. Additionally, the SnO_2_ can be easily manufactured as thin films and powder. According to Cetinorgu et al., vacuum arc-formed SnO_2_ thin films are chemically stable in HCl and NaOH solutions and did not dissolve after 27 h [5]. Moreover, a specific capacity in the order of 1.49 A h.g^−1^ was reported for SnO_2_ nanoparticles (NPs). Additionally, SnO_2_ has a wide direct bandgap (*E_g_* = 3.6 eV at room temperature (RT)), which slows down degradation and increases device stability, but it also reduces the number of photogenerated electron-hole pairs that are generated when exposed to UV radiation. Therefore, it may be used for solar energy applications, such as the production of photoelectrochemical (PEC) H_2_, if it is made with more surface area and optical absorption in the UV/Vis region. Consequently, SnO_2_ is attracting continuous interest for utilisation in optoelectronic devices, sensors, capacitors, Li-ion batteries, UV photocatalytic, and as an electrode material for energy conversion [3,4,6,7,8,9].

The multifunctionality of SnO_2_ can be tuned by controlling the synthetic technique and preparative parameters and by inducing compositional changes via doping processes. Srinivas et al. reported that Co doping can introduce RT ferromagnetism in SnO_2_ as diluted magnetic semiconductors [10]. This ferromagnetism was found to depend on the surface diffusion of Co atoms, defects distribution, size of the material, and its surface conditions. According to Mazloom and Ghodsi, SnO_2_’s n-type conductivity can change to p-type with an increase in the films’ resistivity as long as the Co doping level is higher than 3.0 at.% [11]. Additionally, Korotcenkov et al. demonstrated that a Co doping level in the range of 2–4% was sufficient to create a sufficient concentration of catalytically active centres (Sn atoms and oxygen vacancies) for O_3_ dissociation and reducing gas oxidation, which produced gas sensing effects for SnO_2_ [12]. Podurets et al. [13] fabricated SnO_2_@ZnO, SnO_2_@SnO_2_, and SnO_2_@TiO_2_ of core-shell morphology by atomic layer deposition (ALD). The obtained *E_g_* for SnO_2_ and TiO_2_ shells were only 2.8 and 3.1 eV, respectively, and these values result in interesting photocatalytic activity under UV and visible-light irradiation. Geng et al. [6] fabricated Au-SnO_2_ nanorods/activated carbon by a solvothermal process to yield an energy density as high as 168.9 Wh/kg. Wu et al. [1] developed SnO_2_ (nanodots)/g-C_3_N_4_ (nanosheets) by one-step polymerisation technique and obtained H_2_ production activity about 6.1 times larger than that of g-C_3_N_4_. The nanodots act as a bridge and yield a higher separation rate for the photogenerated electrons. Bozheyev et al. [14] achieved a photocurrent density of 6 × 10^−4^ A/cm^2^ at 1.23 V_RHE_ and the highest incident photon to current transfer efficiency (IPCE) of 22% at 450 nm by controlling the Mo doping ratio in the magnetron co-sputtered SnO_2_ films. Additionally, SnO_2_/BiVO_4_/rGO construction yielded a photocurrent of 2.05 mA/cm^2^ at 1.23 V_RHE_, (about four times of the BiVO_4_ alone) and IPCE of ~2.5 times that of BiVO_4_ at 400 nm [15]. Wang et al. [16] fabricated SnO_2_/Ag/SnO_2_ tri-layers on float substrate by magnetron sputtering. The film showed a sheet resistance of 5.92 Ω/sq and transmittance of 87.0% which is useful for use in CdTe solar cells.

Moreover, interesting modifications in the performance of SnO_2_ were achieved by doping with transition metals. Cu and N co-doping during the hydrothermal synthesis of SnO_2_ induced a large increase in O deficiency from ~27.2 to 36.7%. This change accelerated the rate of charge transfer and improved the performance of SnO_2_ in the reduction of CO_2_ to format a liquid fuel and H_2_ storage material [17]. Ismail and Abdul Majeed [18] deposited Pd-doped SnO_2_ by spray pyrolysis on porous Si substrates to work as a solar cell with a conversion efficiency >14% at 5 wt.% Pd doping combined with V_oc_ = 456 mV, J_sc_ = 0.041 A/cm^2^ and FF = 79%. Sohal et al. [19] prepared Er (1–5 wt.%) doped SnO_2_ NPs by co-precipitation and films of thicknesses 50–150 nm by electron beam evaporation. The 100 nm thick film doped with 3.0 wt.% Er exhibited a maximum response for NO_2_ at RT. Ni and Co co-doped SnO_2_ NPs with crystallite sizes between 12.5 and 22 nm and grains sizes between 50.36 and 78.2 nm were produced by Matussin et al. using a biogenic and ecologically benign approach [20]. These NPs showed enhanced activity as photo-antioxidants and in photoconversion of 4-nitrophenol under visible light irradiation. Similarly, Khan et al. [2] reported the total removal of 4-Nitrophenol in less than 5 min by using the chemically prepared Fe-SnO_2_ NPs. The crystallinity of chemically produced SnO_2_ NPs is increased, the rate at which electron/hole pairs recombine is decreased, and the photocatalytic activity toward RhB, when exposed to visible light, is increased, according to Toloman et al. [8].

The material’s crystallinity is mainly relayed on the heat treatment and this crystallinity affects the *E_g_*, rate of electron-hole recombination, and the efficiency of energy conversion [21]. Based on the literature survey, there is no complete report on the effect of Co doping and heat treatment on structural properties, water splitting ability, and H_2_ production efficiency of SnO_2_ thin films. This work aims to demonstrate for the first time the effects of Co doping and calcination temperature (CT) on the structure, morphology, optical characteristics, and PEC catalytic activity of the spin-coated SnO_2_ photoelectrodes. In addition to examining the impact of PEC temperature on the performance of the optimised photoelectrode, the conversion efficiencies under white and monochromatic light illumination and the number of hydrogen moles generated are also assessed. In particular, this study focuses on optimizing the CT and Co doping level to increase the optical absorption and surface area by enhancing the optical and textural aspects of PEC SnO_2_-based electrodes for solar H_2_ generation.

## 2. Experimental Section

### 2.1. Materials and Preparation

Un-doped and 2.5, 5.0, and 7.5 at.% Co-doped SnO_2_ thin films were prepared by dissolving ~1.13 g SnCl_2_.2H_2_O (Merck, Darmstadt, Germany) of molecular weight (M_W_) = 225.63 g/mol in 10 mL pure ethanol. For the doped solution, certain amounts of Co(NO_3_)_2_. 6H_2_O or CoH_12_N_2_O_12_ (Aldrich, St. Louis, MO, USA) of M_W_ = 291.03 were added. The solutions were stirred at 323 K for 2 h till getting clear and homogeneous solutions and aged for 24 h at (300 K). The glass slides of specific dimensions were treated by sonication in acetone, methanol, and deionised water before being air dried. The sol-gel produced solutions were deposited on the glass slides at 2000 rpm for 25 s, then dried at 473 K for 5 min on an electric hot plate. Six coatings were performed to increase the films’ thickness, before the final calcination at 673, 773, and 873 K for 2 h in an air furnace. In each case, the furnace was left to cool to RT and this process takes 8–10 h. Before use, the samples were kept in zipper bags to avoid the moisture effect.

### 2.2. Measurements

Investigating phase purity and crystallinity was done by using X-ray diffraction (X’PertPro MRD, Philips, Malvern, UK) by assisting with Cu–K_α_ radiation of wavelength 0.1542 nm during steps of 0.02°. 2D and 3D images to study the film’s morphology, roughness, and thickness of the samples were taken by the atomic force microscope of non-contact mode (XE-100E, Park, Suwon, Korea). The vibrational properties of the films (Raman spectra) were recorded by Thermo Fisher (Austin, TX, USA) DXR Raman spectrometer with an 8 mW power laser beam of 532 nm wavelength. The absorbance spectra in the range of 200–1350 nm, for determination of the bandgaps, were recorded by UV-vis-NIR 3700 double beam Shimadzu spectrophotometer (Maryland, MD, USA). All these measurements were carried out at RT. The PEC H_2_O splitting was measured by a 2400-Keithley SourceMeter in 100 mL of 0.5 M HCl, NaOH, and Na_2_SO_4_ solutions at RT with a scanning rate of 1 mV/s. The nanocomposite electrode with a 1 cm^2^ surface area was used as the photoanode, and the counter electrode of the same area was the Pt-electrode. i.e., for PEC measurements, both a working 1 cm^2^ electrode and a counter 1 cm^2^ Pt electrode are used (2-electrode System). The photocurrent density-voltage (J_ph_-V) behaviours were measured under a standard white illuminance (AM 1.5 G, 100 mW/cm^2^) and evaluated with the use of a 400 W mercury xenon light source (Newport, MODEL: 66926-500HX-R07, Newport, UK) and utilizing the 2400-Keithley SourceMeter (Tektronix company, Beaverton, OR, USA).

## 3. Results and Discussions

### 3.1. XRD and AFM Study

Figure S1 (Supplementary Materials) shows XRD patterns of the as-deposited films (before annealing). The wide peak (halo) extends from 17° to 38° on the 2θ scale indicating the amorphous structure of the film. After doping with 2.5–7.5% Co, two peaks around 26° and 34° appear to be superimposed on this halo peak. This indicates that Co doping enhanced the crystallinity of the as-deposited films. This can take place when the Co^2+^ ions form stable solid solutions with SnO_2_ and take the regular lattice positions in the SnO_2_ lattice [22].

**Figure 1:**
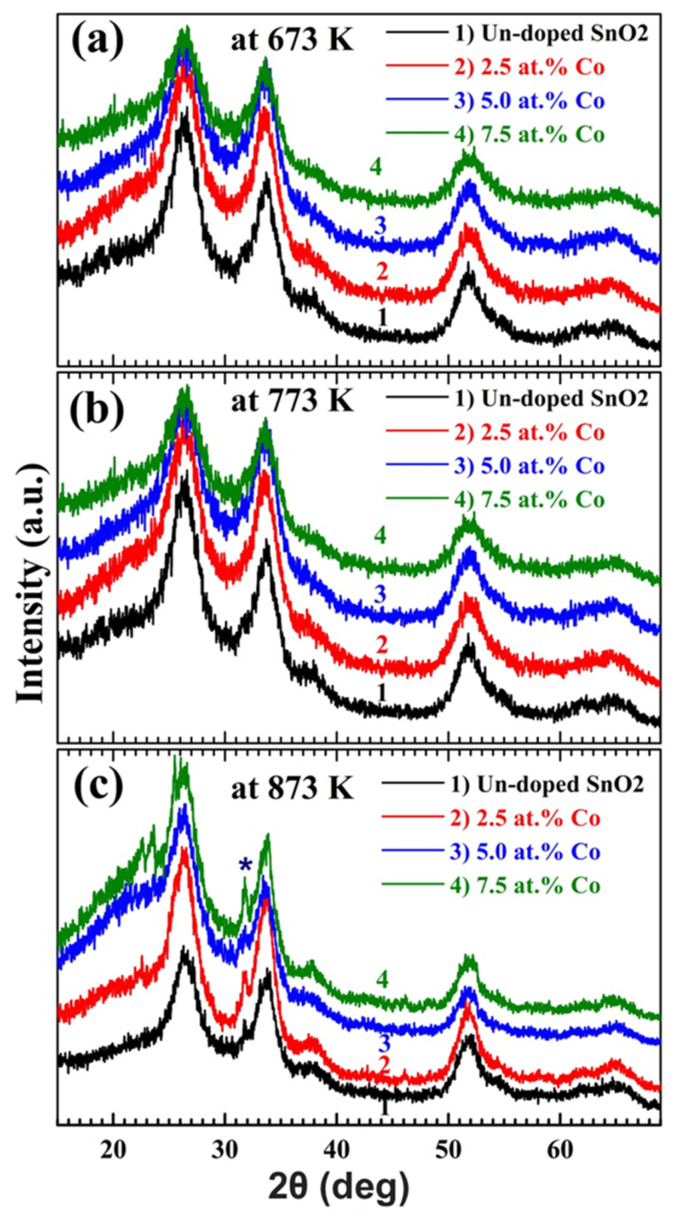
(**a**–**c**) XRD charts of pure and doped tin oxide films at different annealing temperatures. The sign (*) indicates the formation of the SnO phase at 873 K.

XRD of the pure and Co-doped films calcined at temperatures in the range of 673–873 K are depicted in Figure 1a–c. As seen, all films show polycrystalline structures. The observed diffraction peaks at ~26.63°, 33.87°, 37.92°, 51.78°, and 64.72° correspond to (110), (101), (210), (211) and (112) planes of SnO_2_ tetragonal phase, respectively. These wide peaks indicate the formation of nano-crystalline SnO_2_ materials. No peaks related to Co metal or one of its oxides are detected. A similar result was reported for the chemically precipitated Fe-doped SnO_2_ NPs [2]. XRD of spray deposited Pd-doped SnO_2_ contained only one sharp (110) peak [18]. However, increasing the annealing temperature to 873 K encouraged the formation of the unstable SnO phase, indicated by a sign (*) and a peak at 31.85° [23]. Similarly, SnO diffraction peaks were observed among those of SnO_2_ that were prepared by the chemical vapour deposition at 600 °C [24].

The preferred orientation feature can be quantified by the texture coefficient (*TC*) that signifies the texture of a specific plane. The thin film with randomly oriented crystallites presents *TC* = 1, and *TC* > 1 implies the preferred growth, i.e., a larger abundance of crystallites oriented in a (*hkl*) direction. *TC* was calculated considering the main three peaks; (110), (101), and (211), using XRD data and the following equation: $TC\left(hkl\right)=\frac{I\left(hkl\right)/I_{o}\left(hkl\right)}{\frac{1}{Z}\sum I\left(hkl\right)/I_{o}\left(hkl\right)}$ [25], where I(*hkl*) and Io(*hkl*) refer to the relative intensity and the standard JCPDS intensity of the plane (*hkl*), respectively, and Z is the reflections. From Table 1, it is noted that *TC* (110) and *TC* (101) are larger than unity. This suggests the existence of more crystals related to these two peaks. The origins of the noted orientation of the (110) plane can be explained by the periodic band chains theory (PBC) [26]. Previously, it was reported that doping with more than 3.0 wt.% Er in the e-beam evaporated snO_2_ films changed the preferred growth direction from (110) to (302) [19]. The CT (673–873 K) does not affect the peaks’ position. Additionally, it is seen from Figure 1 that the doping with 2.5–7.5 at.% Co causes no peaks’ shift. This may be because the two ions Co^2+^ and Sn^4+^ have very close ionic radii of 0.74 Å and 0.71 Å, respectively [20]. Carbon doping (10–50 wt.%) into SnO_2_ resulted in a slight shift (by 0.26°) towards lower 2θ values [3]. Figure 1a demonstrates that for the undoped film, the calcinated sample at 773 K had more intense XRD peaks than the calcinated sample at 673 K. This suggests that the film’s crystallinity improved when the CT was raised from 673 to 773 K. A similar observation was reported for SnO_2_ NPs prepared by continuous flow hydrothermal route, at pH = 3 and annealing temperatures in the range of 523–673 K [7]. However, increasing the temperature to 873 K made the peaks of reduced intensity. This may ascribe to the mismatching between the film and the glass substrates which may be bent at this high temperature. SnO_2_ film doped with 2.5 at.% Co and calcined at 673 or 773 K exhibits peaks of relatively higher intensity compared with those of the un-doped film indicating an additional increase in the film’s crystallinity. Increasing Co content to 5.0 and 7.5 at.% reduces the peaks’ intensity of the film calcined at 673 K, but this intensity increases with increasing temperature to 773 and 873 K. The broadness of SnO_2_ diffraction lines with increasing Co content suggests the intercalation of Co^2+^ into SnO_2_ and these ions create specific disorders and lead to compositional heterogeneity [2].

The crystallite size (*C_s_*) and the dislocation density ($\delta =\frac{1}{{C_{s}}^{2}}$) of the samples were calculated utilizing the Scherer equation; *C_s_* = *0.9λ/Γ cos θ,* where λ = 0.154 nm (the wavelength emitted from the *K*_α_-Cu source), *Γ* is the full-width at half maximum intensity, considering the main three peaks; (110), (101) and (211). The obtained values are listed in Table 1. As can be noticed, Co doping and CT have a significant effect on both *C_s_* and *δ*. All films have *C_s_* in the range of 18.4–29.2 nm. Higher content of Co (5.0–7.5 at.%) leads to a decrease in *C_s_*, however, increasing CT hinders this increase. Similarly, excessive doping with Ta (2.6–15.5 at.%) resulted in deterioration of crystallinity of SnO_2_ film prepared by radio-frequency sputtering [9]. Er-doping significantly increased the crystallite size of SnO_2_ films from 17.3 nm to ~29.5 nm, which was then marginally changed with increasing Er content [19]. The obtained results mean that the films’ crystallinity can be controlled by Co doping and calcination.

**Figure 2:**
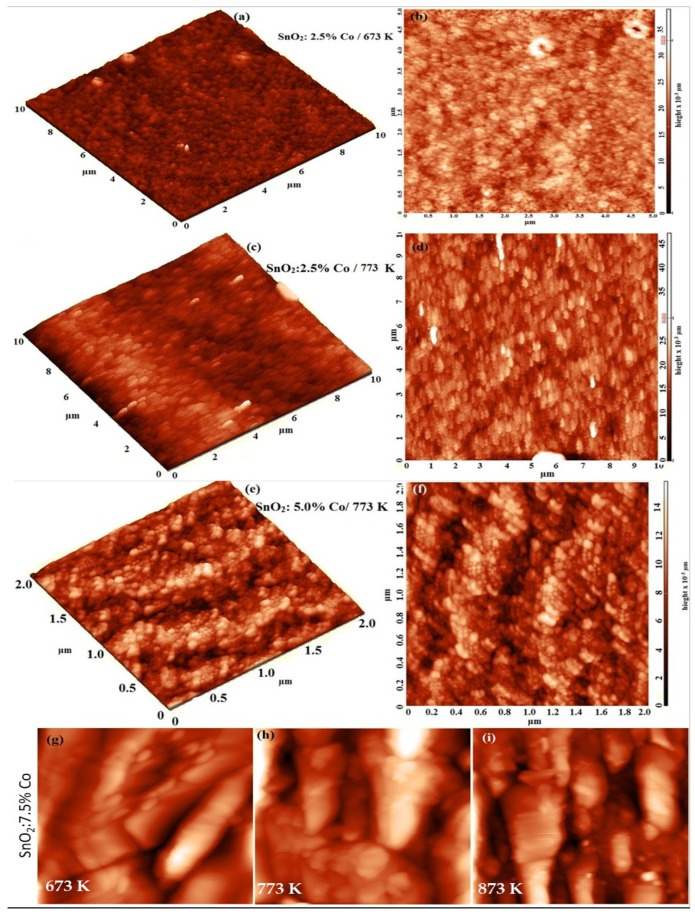
3D & 2D AFM images of (**a**–**d**) SnO_2_: 2.5% Co at 673 K and 773 K, (**e**,**f**) SnO_2_: 5.0% Co at 773 K; and (**g**–**i**) 2D AFM images of SnO_2_: 7.5 thin films at 673, 773,and 873 K, respectively.

**Figure 3:**
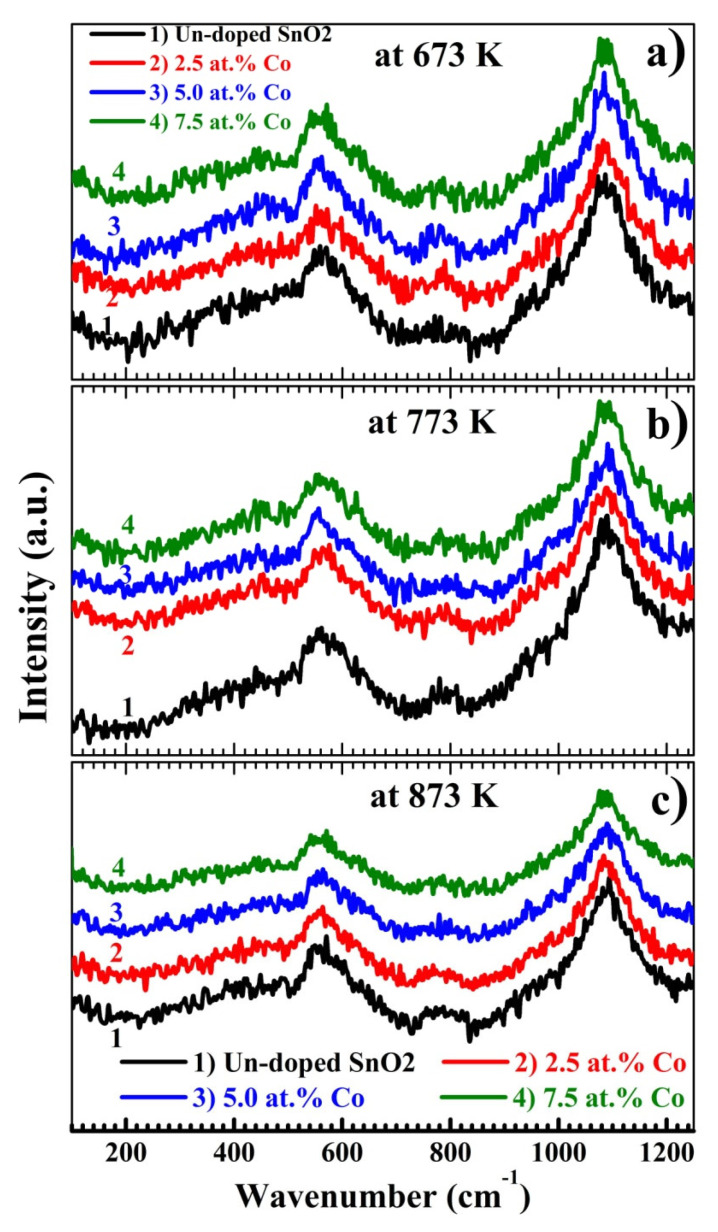
(**a**–**c**): Raman spectra of pure and doped tin oxide films at different CT.

The films’ surface morphology was examined by AFM via capturing 2D and 3D images, Figure 2a–f and Figure S2 (Supplementary Materials). As seen from Figure S2, the unannealed pure film exhibits no definite morphology, however, the 5.0% Co doped film exhibits a nanoparticulate morphology with a relatively high particle density per unit area. The 3D and 2D images of 2.5 at.%-doped SnO_2_ calcined at 673 K, Figure 3a,b illustrates that the surface is of granular morphology, i.e., the surface is composed of a very large number of spherical-shaped particles and size less than 20 nm and average root-mean-square roughness (R_rms_) of ~18 nm. Increasing the CT to 773 K led to a larger grain size of 23 nm and reduced R_rms_ to 14 nm, Figure 2c,d. However, increasing Co content to 5.0 at.% decreased the grain size to 22 nm and the R_rms_ to 11 nm, Figure 2e,f. The obtained thicknesses of the films were ranged from 255 to 270 nm, as mentioned in Table 2. Thus, the prepared films are of relatively smooth surfaces and are useful for optoelectronic and photocatalytic applications [9]. Figure 2g–i shows the role of CT (673–873 K) on the SnO_2_ films doped with the higher content of Co (7.5 at.%). As seen, both the grain size and surface roughness increase with annealing temperature. Similarly, the chemically precipitated Fe-SnO_2_ appeared under FE-SEM in the form of blocks of 500 nm in size that were composed of NPs with an average size of ≈25 nm [2]. These findings show that the measurements made by XRD and AFM are consistent.

### 3.2. Raman Spectral Analysis and Optical Properties

For deeper insights and further confirmation of the SnO_2_ phase under calcination and Co loadings, Raman spectra were recorded and presented in Figure 3. Four peaks appear in the spectra of the films; two weak peaks at 452 and 780 cm^−1^ and two strong wide peaks at 552 and 1090 cm^−1^. The 452 cm^−1^ peak (*E_g_* mode) and 780 cm^−1^ peak (B_2g_ mode) are due to the vibration of O in the O plane and the construction/expansion of Sn–O bonds, respectively, which usually occur in nanocrystalline tin oxide [25]. B_2g_ mode is slightly shifted under the effect of CT and Co loading. The broad peak at 552 cm^−1^ is related to the rutile phase of SnO_2_ [19]. This result is consistent with XRD results. As seen, increasing the CT and Co content resulted in changing the intensity of this peak. The most intense broad peak at 1090 cm^−1^ is related to the multi-phonon transitions [27]. The *E_g_* mode is highly sensitive to oxygen vacancies, and its appearance indicates that our samples can be used for photocatalytic applications and photoelectrochemical cells.

**Figure 4:**
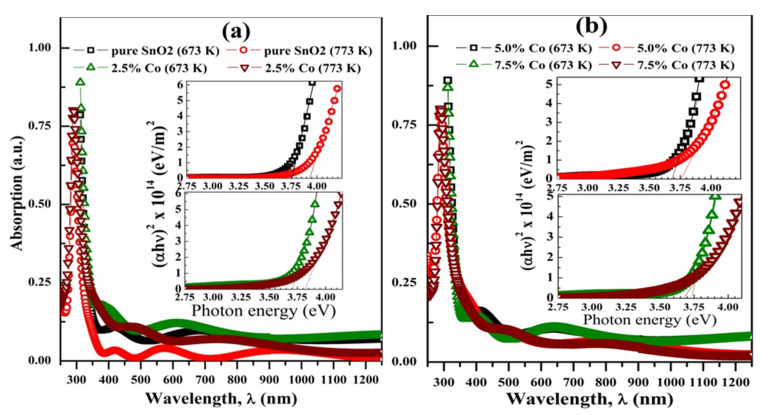
Optical absorption spectra of (**a**) pure and 2.5 at.%, and (**b**) 5.0 at.% and 7.5 at.% Co-doped tin oxide films at 673 and 773 K. The insets are Tuac’s plots for bandgap determination.

Figure 4a–d displays the UV/Vis absorbance spectra of the films doped with 0.0–7.5 at.% Co and calcined at 673 and 773 K. The films exhibit an absorption peak at ~290 nm and an absorption edge at ~235 nm which is equivalent to 3.70 eV. According to Puga et al. [28], SnO_2_ exhibits a substantial UV absorbance band (270–300 nm). The clear interference fringes that are seen in all films confirm the smoothness and homogeneity of the films seen in AFM images. The 2.5% Co-doped film annealed at 673 K shows relatively higher absorption in the studied wavelength range. The interference fringes of films calcined at 773 K begin at a lower wavelength and their number is larger than the films calcined at 673 K. Increasing CT resulted in denser films of more compacted layers. The direct optical band gap *E_g_* can be evaluated utilizing Tauc’s formula (*αhυ*)^2^ = *A*(*hυ–E*_g_), whereas the incident photon energy $h\upsilon \;\left(\mathrm{eV}\right)=\frac{1242}{\lambda \;\left(\mathrm{nm}\right)}$, *A* is a constant, and *α* is the absorption coefficient (*α* = 2.303 Absorption/film thickness). The insets of Figure 4a,b show the (*αhυ*)^2^ vs. *hυ* plots. *E_g_* is obtained by extrapolating the linear portion to zero absorption and Table 2 provides their values. *E_g_* values are between 3.67 and 3.93 eV. The decrease in *E_g_* of SnO_2_ with doping till 5.0 at.% is related to the introduction of electronic states in its bandgap. Although it has been reported that increasing CT increases oxygen vacancies [29], the effect of enhanced crystallisation outweighs the effect of oxygen vacancy concentration (Ov), which results in a decrease in the number of charge carriers inside the material in films that are calcined at 773 K. The drop in *E_g_* from 3.73 to 3.67 eV at a doping level of 7.5 at.% may be related to an elevated Ov. This is also anticipated at CT = 873 K, where the presence of the low valence state (Sn^2+^), depicted in Figure 1c, results in an increase in Ov and unbound electrons that lower the conduction band’s bottom and, consequently, reduce *E_g_*. In rare circumstances, the Ov can introduce mid-gap states by acting as shallow donors [30].

Therefore, Co doping and calculations can be effectively used to tune the optical properties of SnO_2_. Yang et al. [17] reported a decrease in the *E_g_* of SnO_2_ from 3.45 to 3.33 eV after doping with 7.0% (Cu and N). Then, 18 at.% Mo doping into SnO_2_ prepared by the magnetron co-sputtering technique shrank the *E_g_* from 3.65 to 2.7 eV [14]. Additionally, 10 at.% (Co, Ni) doping reduced the *E_g_* of SnO_2_ NPs synthesized using a green process from 3.33 to 2.08 eV [20]. Moreover, Pd doping (1–5 wt.%) narrowed *E_g_* of SnO_2_ from 3.65 to 3.25 eV [18]. Ta doping in the range of (2.6–7.8 at.%) widen *E_g_* of SnO_2_ from 3.99 to 4.21 eV that then reduced it to 4.17 eV at 15.5 at.% Ta. In addition, Er doping (0–3 wt.%) for the e-beam evaporated SnO_2_ film, 100 nm thickness, narrowed its *E_g_* from 3.88 to 3.63 eV, then it widen to 3.72 eV at 5.0 wt.% Er [19]. Thus, doping with transition metal is a facial method to reduce *E_g_* of SnO_2_ to be more valid for photocatalytic application under visible light exposure.

### 3.3. Photoelectrochemical H_2_ Generation

#### 3.3.1. Influence of the Doping Ratio and the Used Electrolyte

**Figure 5:**
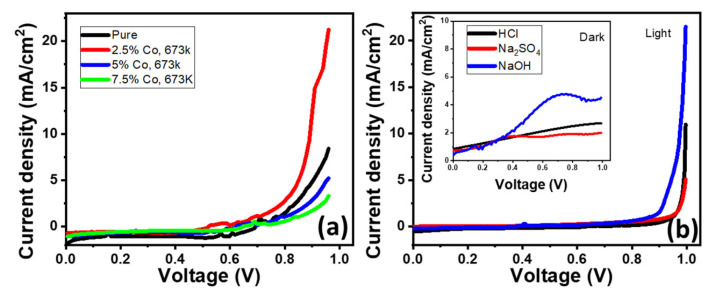
(**a**) The electrodes’ photocurrent density-voltage curves in 100 mL of 0.5 M NaOH for different samples, and (**b**) The (2.5% Co, 673 K) electrode photocurrent density-voltage curves in 100 mL of 0.5 M HCl, Na_2_SO_4,_ and NaOH for optimised sample in the existence and absence of light.

The PEC cell electrolyte is one of the essential things that has a major impact on the PEC performance of the photoelectrode. The photocatalytic interfaces will differ in several ways depending on how the electrolytes are changed. The chosen electrolyte must be compatible with the PEC catalyst being used. Electrochemical interactions between photo-electrodes and electrolytes are prohibited, and their optical absorption bands must not overlap. The electrolyte structure, which alters the conductivity of electrolytes and affects ion transport, has a substantial impact on the rate of PEC H_2_ generation. The rate of H_2_ oxidation and the catalytic effectiveness of the PEC are influenced by the electrolyte’s conductivity and pH [31]. The electrolyte impedance may decrease due to insufficient active cations or anions like Na+/SO_4_^2−^ or OH^−^, which may stimulate band bending. To pinpoint the main differences in the PEC performances of the examined photoanodes; a strong acid (HCl, pH = 0.3), a strong basic (NaOH, pH = 13.69), and a neutral salt (Na_2_SO_4_) were all used at the concentration (0.5 M). In Figure 5b, the current density rises in the following order: J_ph_(NaOH) > J_ph_(HCL) > J_ph_(Na_2_SO_4_), with the J_ph_’s value growing as the utilised bias rises. The maximum J_ph_ value in solutions of NaOH, HCL, and Na_2_SO_4_ is 21.25 mA/cm^2^, 10.972 mA/cm^2^, and 5.09 mA/cm^2^, respectively. Therefore, NaOH is the ideal electrolyte for the PEC process using 2.5% Co-doped SnO_2_ anode calcination temperature at 673 K, abbreviated to (2.5% Co, 673 K) SnO_2_. By reducing the Fermi level to a lower energy level through adsorbing extra H+/OH− species following the relationship E_F_ = E_F_(pH_0_) − 0:059 × pH (at RT), the surface charges of doped metal oxides and the separation of electrons from holes are both influenced by pH variations [32]. In other words, depending on the pH of the electrolyte solution, the fermi level for metal oxide semiconductors is located at various energy sites. The location of the Fermi level at the electrode’s surface has an impact on how much the potential of the photo-generated electrons is reduced. The overpotential or motive factor for hydrogen synthesis reduces as E_F_’s energy level rises [33]. The J_ph_’s value for the (2.5% Co, 673 K), SnO_2_ photoelectrode (21.25 mA/cm^2^) is significantly high than that for the (0% Co, 673 K), and (7.5% Co, 673 K), SnO_2_ photoelectrodes (8.43, 5.23, and 3.325 mA/cm^2^, respectively) under light exposure. despite the very slight difference in energy gap between them. Due to the surface’s composition of many spherical particles with sizes less than 20 nm and higher R_rms_ (18 nm) than other samples, it exhibits more absorption in the wavelength range under study and has a larger surface area. In order to get the highest photocurrent density in the NaOH electrolyte, the optimum photoanode is (2.5% Co, 673 K) SnO_2_. With the addition of OH^−^ ions in that pH range, the measured current density in the various electrolytes increases at pH ≥ 7.

#### 3.3.2. PEC Reusability and Stability of the (2.5% Co, 673 K) SnO_2_ Photoanode

**Figure 6:**
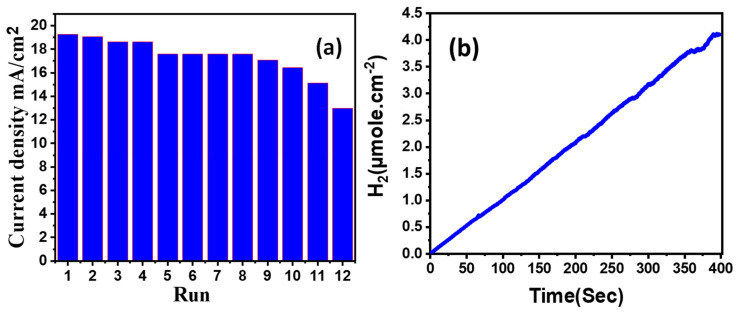
The change of J_ph_ of (2.5% Co, 673 K) photoelectrode (**a**) for 12 runs of reusability under white light illumination at +1 V in RT and (**b**) the number of H_2_ moles with elapsed time for (2.5% Co, 673 K) photoanode @+1 V in RT.

In 0.5 M NaOH and utilizing a 2400-Keithley SourceMeter the reproducibility of the (2.5% Co, 673 K) SnO_2_ photoanode was investigated by measuring the photocurrent density at +1 V in a standard white light environment for 12 consecutive runs, as illustrated in Figure 6a. The photocurrent density of the (2.5% Co, 673 K) SnO_2_ photoanode was 19.28 mA/cm^2^ in the first run, indicating that the photoelectrochemical water splitting mechanism is more efficient in light, as after 12 consecutive cycles, the photocurrent density decreased by 32.76%, from 19.28 to 12.99 mA/cm^2^, showing excellent reproducibility and reusability for the optimised SnO_2_ photoanode in white light at RT.

The PEC stability of the (2.5% Co, 673 K) photoanode is studied for a lengthy time in 0.5 M NaOH under the influence of white light and @+1 V between the photoanode and the platinum electrode. The change in photocurrent density with time is used to calculate the number of PEC H_2_ moles as shown in Figure 6b [34]. Equation (1) was theoretically used to calculate the number of H_2_ moles using Faraday’s law [35];

(1) $$H_{2}(\mathrm{moles})=\;\int_{0}^{t}\frac{J_{\mathrm{ph}}\mathrm{dt}}{F}$$

where F = 9.65 × 10^4^ C/mol (Faraday constant) and t is the elapsed time during the process. The number of hydrogen moles produced over time is presented in Figure 6b. This figure almost showed linear production of hydrogen, which can be used to confirm the stability of the designed PEC electrode. The obtained number of H_2_ moles per active area theoretically is 41.4 mmol h^−1^cm^−2^. To measure the flow rate of the H_2_ gas in SCCM, the outlet of the closed PEC unit is connected to the ALICAT Mass Flow Meter (Model: MB, 0-100SCCM (Standard mL/min)). Experimentally, the value is found to be 14.71 SCCM for our 1 cm^−2^ film which is equivalent to 39.45 mmol.h^−1^.cm^−2^. This value, which is pretty close to the value obtained theoretically, confirms the excellent effectiveness of the proposed photoelectrode.

The solar-to-hydrogen efficiency (STH), which is the overall efficiency of the PEC water splitting cell, is defined as the proportion of overall hydrogen energy production to overall solar energy (AM 1.5 G, 100 mWcm^2^). Equation (2) was used to calculate STH % [36]:

(2) $$\mathrm{STH}=[(H_{2}/S)\;\times \;(237\;\mathrm{KJ}/\mathrm{mol})]/[P_{\mathrm{total}}\;\times \;A]$$

where P_total_ is the illuminating light’s total power density in mW/cm^2^, *A* is the lighted section of the photoelectrode’s area in cm^2^, and H_2_/S is the H_2_ moles’ generation rate in mmol/sec. The computed STH for the (2.5% Co, 673 K) photoelectrode is 24.65%.

#### 3.3.3. Effect of Temperature and Thermodynamic Parameters

**Figure 7:**
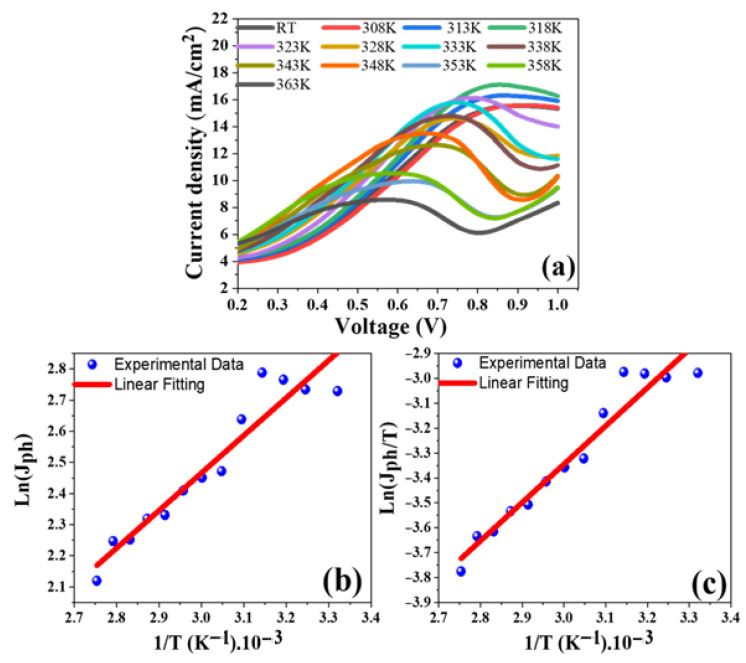
Effect of temperature for the 2.5% Co, 673 K; (**a**) the change of J_ph_-voltage curves at distinctive temperatures, (**b**) ln(J_ph_) vs. (T^−1^), and (**c**) Ln(J_ph_.T^−1^) vs. (T^−1^).

Figure 7a shows how the PEC J_ph_-voltage curve of the (2.5% Co, 673 K) photoelectrodes in 0.5M NaOH electrolyte was affected by heating from RT to 363 K. The current density increased from 15.4 mA.cm^−2^ to 17.1 mA.cm^−2^ by rising T to 318 K and then reduced from 15.4 mA.cm^−2^ to 8.3 mA.cm^−2^ by raising T to 363 K, as can be seen in this figure. Evaluation of thermodynamic parameters including activation energy (*E*_a_), enthalpy (ΔH*), and entropy (Δs*) is also required. Figure 7b illustrates the relationship between the absolute temperature reciprocal (1/T) and the J_ph_ (rate of reaction) of the photoelectrode of (2.5% Co, 673 K). The slope of the linear fitting in Figure 7b is used to calculate the value of *E*_a_ using the Arrhenius equation [37]:

(3) $$\mathrm{Ln}\;\left(J_{\mathrm{ph}}\right)=-\frac{E_{a}}{R}$$

where *R* is the universal gas constant (8.314 J/K.mol). From Figure 7b, slope = −*E*_a_/*R* and the *E*_a_ of the (2.5% Co, 673 K) electrode is 10.032 kJ/mol. In addition, the values of ΔH* and ΔS* for the PEC H_2_ production are estimated by using Eyring equation by drawing a relationship between Ln (J_ph_.T^−1^) and (T^−1^) as displayed in Figure 7c. The Eyring equation can be written as [38]:

(4) $$\mathrm{Ln}\frac{J_{\mathrm{ph}}}{T}=-\frac{{\Delta H}^{*}}{R}.\;\frac{1}{T}+\mathrm{Ln}\left(\frac{K_{B}}{h}\right)+\frac{{\Delta S}^{*}}{R}$$

where *K*_B_ = 1.38 × 10^−23^ J/K (the Boltzmann’s constant), and *h* = 6.626 × 10^−34^ J.s (the Planck’s constant). For (2.5% Co, 673 K), the linear fitting’s slope gives a value of 12.78 kJ/mol for ΔH*, while its intercept gives a value of 311.113 J/mol for ΔS*.

#### 3.3.4. Effect of Monochromatic Light Illumination and Conversion Efficiencies

**Figure 8:**
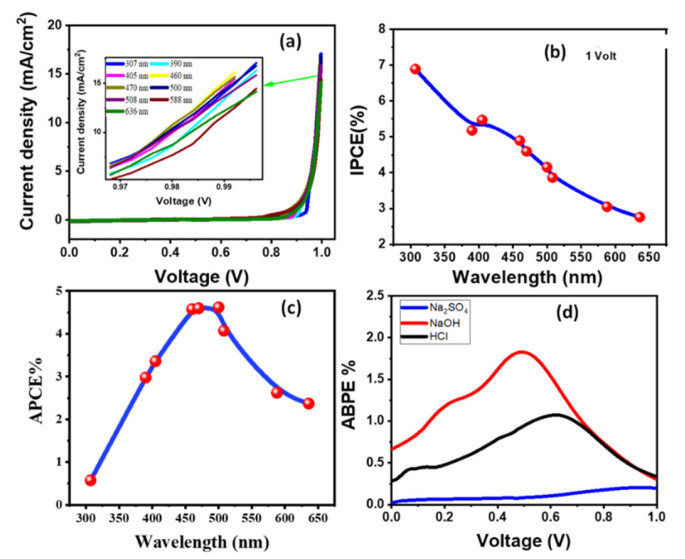
(**a**) J_ph_-voltage characteristics of (2.5% Co, 673 K) photoelectrode under monochromatic light illumination at RT in 0.5M NaOH, (**b**) IPCE% and (**c**) ABCE% (λ) @+1 V versus the incident wavelength, and (**d**) ABPE% versus the applied voltage for various electrolytes.

The J_ph_ is shown vs. applied voltage for the (2.5% Co, 673 K) photoanode in 0.5M NaOH at room temperature under monochromatic light in Figure 8a. A series of optical bandpass filters covering the visible spectrum from 307 to 636 nm were used. The maximum photocurrent, J_ph_ = 17.06 mA.cm^−2^, was obtained at 307 nm. The lowest photocurrent, J_ph_ = 14.14 mA.cm^−2^, was recorded at 636 nm as illustrated in the inset of Figure 8a. This current density-wavelength dependency may be related to the photo electrocatalytic response of the optimum photoelectrode for the H_2_ production process and supports the absorption characteristic of the (2.5% Co, 673 K) photoelectrode in every wavelength. In general, this behaviour supports the photoelectrode’s comprehensive response and ability to absorb a significant amount of the solar spectrum in the UV-Vis range.

Measuring the incident photon-to-current conversion efficiency (IPCE) at various wavelengths further ensures the improved solar absorption of the (2.5% Co, 673 K) photoelectrode and its use for effective H_2_ generation from H_2_O splitting. Equation (5) is used to get the IPCE% at a constant applied voltage (+1 V) [39,40]:

(5) $$\mathrm{IPCE}\%=1240.\frac{J_{\mathrm{ph}}}{\lambda .P}.\;100\;$$

where *λ*(nm) is the wavelength of the incident photons and *P* is the incident light power density of the Xenon lamp (Newport, 66142-500HX-R07) at each wavelength. Figure 8b represents the fluctuation of IPCE percentage with wavelength (λ). The (2.5% Co, 673 K) photoelectrode’s highest IPCE % is 6.892% @307 nm, which is in line with the optimum photoelectrode’s maximal absorption. Optically related losses such as photon transmittance (Tr) and reflectance (R) were not taken into account in the IPCE calculation. To account for optical losses, it is required to assess the internal quantum efficiency, also referred to as the absorbed photon-to-current conversion efficiency (APCE%). The percentage of PEC-produced charges that contributed to the resulting photocurrent per absorbed photon is known as the APCE%. APCE is calculated by Equation (6) [8,41]:

(6) $$\mathrm{APCE}(\lambda )=\frac{\mathrm{IPCE}\left(\lambda \right)}{A\left(\lambda \right)}=\frac{\mathrm{IPCE}\left(\lambda \right)}{1-R-T_{r}}$$

where A(*λ*) is the optical absorbance. Figure 8c demonstrates the change of APCE% versus the incident *λ*. This figure shows three prominent APCE% values; 4.61% at ~500 nm, 4.595% around 470 nm, and 4.575% around 460 nm.

The extra electrical energy to the system must be removed when introducing a tiny applied voltage to the PEC system to estimate the photoelectrode catalytic efficacy. The applied bias photon-to-current efficiency (ABPE%) might be employed for this purpose. Equation (7) is used to calculate the ABPE% values for the designed photoelectrode [42,43]:

(7) $$\mathrm{ABPE}\%=J_{\mathrm{ph}}\times \frac{\left(1.23-V_{app}\right)}{p}\times 100,$$

where 1.23 is the standard state reversible potential of H_2_O and the externally applied voltage is denoted by Vapp. Figure 8d shows the ABPE % values for various electrolytes as a function of applied voltage. The highest value of ABPE% is 1.827%@NaOH 0.495 V, ABPE % of 1.07% @HCL 0.619 V and 0.19% @Na_2_SO_4_ 0.94 V, respectively. It is worth noting that the photoelectrode’s good performance at low voltage might be beneficial for PEC cell setup.

#### 3.3.5. PEC Impedance Spectroscopy (PEC-IS)

**Figure 9:**
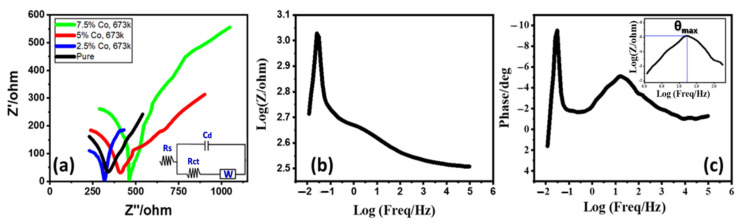
(**a**) Nyquist plots for undoped and doped photoelectrodes in 0.5M NaOH electrolyte at 298 K and 0 V (vs. Ag/AgCl) and under white light illumination. Bode plots (**b**) the change of the overall impedance versus frequency and (**c**) the change of phase versus frequency.

The charge transfer between the active photoelectrode and the electrolyte junction determines the photoelectrochemical system’s impedance. PEC-IS study was made at room temperature using CHI electrochemical workstation (CH Instruments CHI660E) to investigate the dynamics of the charge carriers for the optimum (2.5% Co, 673 K) photoelectrode. The PEC-IS measurements’ frequency (*f*) range was 0.01–100,000 Hz at 0 V (vs. Ag/AgCl) under white light exposure. Figure 9a displays Nyquist plots of electrodes submerged in 0.5M NaOH at (0.0% Co, 673 K), (2.5% Co, 673 K), (5.0% Co, 673 K), and (7.5% Co, 673 K). The Bode charts in Figure 9b,c show the outcomes for the optimum (2.5% Co, 673 K) electrode. The redox reaction is shown by a narrow semicircle of a modest diameter on the high-*f* range of the (2.5% Co, 673 K) photoelectrode Nyquist plot [44], which is subsequently a linear stage filling the area of middle/low *f*s. The middle/low *f*s area is covered by a linear transition that follows the redox process [44]. These PEC-IS plots demonstrated mixed diffusion/kinetic controlled pathways. To further comprehend the PEC-IS observations via hydrogen evolution reaction (HER), the PEC-IS spectra were fitted to a straightforward analogous Randle circuit in the inset of Figure 9a. Using the ZSimpWin program, the Randle equivalent circuit was applied to simulate the PEC-IS spectra, as illustrated in Figure 9a. From Nyquist plot intercepts, the electrolyte resistance (R_s_ = 171 Ω) is obtained at high *f*s. The semicircle diameter in the Nyquist plot is the same as the charge transfer resistance (R_ct_ = 145 Ω). Concurrently, in the analogous circuit, the Warburg impedance is W = 0.0092 and the double-layer capacitance is C_dl_ = 1.99 mF. The fundamental regulator of the HER is thus the charge transfer process (CTP), which is ensured by the uni-loop of the Nyquist plot. The optimum electrode can then generate a significant quantity of H_2_. The electron/hole recombination is typically deployed in conjunction with the charge transfer procedure (CTP) to regulate the HER. In other words, the small value of R_ct_ that was observed indicates that the amount of charge recombination at the electrode/electrolyte interface was much decreased, which is consistent with the enhanced HER [45]. Bode charts for the (2.5% Co, 673 K) photoelectrode at the H_2_ generation possibility in 0.5 M NaOH electrolyte at 25 °C are displayed in Figure 9b,c. Figure 9b depicts the overall impedance variation with *f*, and Figure 9c shows the phase variation with *f*. This chart illustrates capacitive contribution in between, along with resistive regimes at low and way higher *f*s. The double-layer capacitance (C_dl_) of the photoelectrode and the charge transfer resistance (R_ct_) relate to the low-*f* regime. The creation of a partially protective layer at the electrode’s surface may be connected to the very high-*f* regime. At 16.63 Hz, the highest phase shift (ϴ_max_ = 5.11°) is discovered. Figure 9c was used to estimate the charge carrier lifetime using the relation $\tau_{n}$ = 1/2π ƒ_max_ [46]. The obtained lifetime for the optimized photoelectrode is 9.57 ms. In addition, the data derived from Figure 9 show a significant decrease in charge recombination at the electrolyte/electrode interface. Along with enhanced ionic conductivity and electrolyte diffusion in the (2.5% Co, 673 K) photoelectrode, this also relates to a kinetically simple PEC system.

Finally, the PEC performance was obtained in this study compared with many previously studied SnO_2_- based PEC electrodes as revealed in Table 3 [47,48,49,50,51,52,53,54,55,56,57,58]. The values of J, IPCE, and ABPE confirm that the (2.5% Co, 673 K) SnO_2_ is an efficient electrode for the PEC H_2_O splitting under visible light exposure. Therefore, it has been concluded that the (2.5% Co, 673 K) SnO_2_ electrode is very suitable for the PEC reactor.

## 4. Conclusions

The influence of Co doping (0.0–7.5 at.%) and calcination temperatures in the range of (673–873 K) on the structure, morphology, optical characteristics, and PEC performance of the sol-gel spin-coated SnO_2_ thin films photoanodes have been studied. The production of the SnO_2_ rutile phase with crystallite sizes in the range of 18.4–29.2 nm was validated by XRD and Raman spectra. The granular structure and smooth surfaces with minimal roughness were revealed by AFM. UV-vis spectroscopy showed that the absorption of the films depends on Co content and annealing temperature, 2.5 at.% Co-doped film calcined at 673 K showed a higher absorption. Additionally, the optical bandgap (*E_g_*) of all films was in the range of 3.67–3.93 eV and significantly depend on Co content and the calcination temperature. After adjusting the sample doping ratio, employing electrolyte (HCl, Na_2_SO_4_, NaOH), electrode reusability, applied temperature, and monochromatic illumination, the produced samples were used for efficient photoelectrochemical hydrogen production from water. Electrode stability, thermodynamic characteristics, conversion efficiencies, amount of hydrogen moles, and PEC impedance were also assessed and discussed. The 2.5% Co-doped SnO_2_ photoanode that annealed at 673 K had the highest photocurrent (21.25 mA/cm^2^), number of hydrogen moles (20.4 mmol/h.cm^2^), IPCE (6.892%@307 nm), and APCE (4.61% at ~500 nm). The optimised photoelectrode may be appropriate for industrial applications due to its excellent stability, high conversion efficiency, and inexpensive cost.

## Figures and Tables

**Table 1:** XRD data: calcination temperature CT, the texture coefficient (*TC*) for (110), (101), and (211) planes, crystallite size Cs and dislocation density *δ*.

Film	CT	*TC*			*C*s (nm)	*δ* × 10^−3^ (1/nm^2^)
		(110)	(101)	(211)		
**Un-doped SnO_2_**	673 K	1.377	1.054	0.568	23.4	1.83
	773 K	1.357	1.062	0.581	29.2	1.17
	873 K	1.267	1.09	0.642	24.3	1.69
**2.5 at.% Co**	673 K	1.368	1.091	0.541	25.6	1.53
	773 K	1.303	1.115	0.582	27.7	1.3
	873 K	1.354	1.096	0.55	26.2	1.46
**5.0 at.% Co**	673 K	1.309	1.139	0.552	21.2	2.22
	773 K	1.319	1.102	0.579	22.3	2.01
	873 K	1.514	1.077	0.41	24.1	1.72
**7.5 at.% Co**	673 K	1.354	1.096	0.55	18.4	2.95
	773 K	1.34	1.115	0.545	22.6	1.96
	873 K	1.489	1.071	0.441	26.7	1.4

**Table 2:** Values of Films’ thickness and optical band gap (*E_g_*).

Film	CT	Thickness (nm)	*E_g_* (eV)
**Un-doped SnO_2_**	673 K	256	3.75
	773 K	259	3.93
	873 K	255	–
**2.5 at.% Co**	673 K	262	3.7
	773 K	265	3.82
	873 K	261	–
**5.0 at.% Co**	673 K	267	3.68
	773 K	270	3.75
	873 K	264	–
**7.5 at.% Co**	673 K	257	3.73
	773 K	259	3.67
	873 K	256	–

**Table 3:** Comparison of the values of J_ph_, IPCE%, and ABPE% for the current work with previously studied values for different SnO_2_-based photocatalysts.

Catalyst	Electrolyte	J_ph_ (mA/cm^2^)	IPCE %	H_2_ Production Rate	Ref.
SnO_2_ decorated tungsten oxide doped TiO_2_ nanotube (Sn-WTNT)	KOH	0.12@1.5 V	-	-	[47]
SnO_2_	Na_2_SO_4_	0.1@1 V	2.3%@400 nm	-	[48]
Cu_2_O/SnO2/RuO_2_	Na_2_SO_4_	−4.25@0.6 E/V vs. RHE	-	-	[49]
Laser-Drilled Fluorine-doped Tin Oxide covered Quartz Electrodes	Sodium Phosphate Buffer	7.8@2.5 V	-	-	[50]
g-C_3_N_4_/SnO_2_	Na_2_SO_4_	0.056@1.2 V	-	-	[51]
Indium Tin Oxide (ITO)/Cr-doped-TiO_2_	H_2_SO_4_	0.572@1.5 V	-	-	[52]
SnO_2_/CdS quantum dots	Na_2_SO_3_	9.9@0 V	40%@375 nm	-	[53]
SnO_2_-ZnO Quantum Dots/g-C_3_N_4_ (SZ/g-C_3_N_4_)	5% aqueous glycerol solution	-	-	13,673.61 μmol g^–1^/5 h	[54]
SnO_2_-g—C_3_N_4_	Na_2_SO_4_	8.9@1 V	-	-	[55]
(1%CuO, 1%CoO) co-doped SnO_2_-TiO_2_	Water/methanol solution (1:1)	-	-	1486.4 µmol g^−1^ h^−1^	[56]
Au–SnO_2_	Na_2_SO_4_	1.3 mA@−1 V	-	-	[57]
SnO_2_/SiC nanowires	H_2_SO_4_	62.0@0.6 V	-	274 μmol g^−1^ h^−1^	[58]
(2.5% Co, 673 K) SnO_2_	0.5M NaOH	21.25@1V	6.892%@307 nm	39.45 mmol.h^−1^ cm^−2^	This work

## Data Availability

Not applicable.

## Supplementary Materials

The following supporting information can be downloaded at: https://www.mdpi.com/article/10.3390/ma15196534/s1, Figure S1: shows XRD patterns of the as-deposited films (before annealing); Figure S2: 2D AFM images for (a) pure and (b) 5.0% Co-doped films before annealing.
