# Supplementary material for: The Impact of Co Doping and Annealing Temperature on the Electrochemical Performance and Structural Characteristics of SnO2 Nanoparticulate Photoanodes
Source: Materials (Basel). 2022 Sep 21;15(19):6534. doi: 10.3390/ma15196534 (PMC9572947; doi:10.3390/ma15196534)
Supplement: Supplementary file 1 [file materials-15-06534-s001.zip › materials-1850838-SI.pdf]

Supplementary Data

The Impact of Co Doping and Annealing Temperature on the Electrochemical Performance and Structural Characteristics of SnO_2 Nanoparticulate Photoanodes

Abeer S. Altowyan [1,*], Mohamed Shaban [2,3,*], Khaled Abdelkarem [3] and Adel M. El Sayed [4,*]

[1] Department of Physics, College of Science, Princess Nourah bint Abdulrahman University, P.O. Box 84428, Riyadh 11671, Saudi Arabia
[2] Physics Department, Faculty of Science, Islamic University of Madinah, P.O. Box 170, Al Madinah Al Monawara 42351, Saudi Arabia
[3] Nanophotonics and Applications (NPA) Lab, Department of Physics, Faculty of Science, Beni-Suef University, Beni-Suef 62514, Egypt
[4] Physics Department, Faculty of Science, Fayoum University, El Fayoum 63514, Egypt
* Correspondence: asaltowyan@pnu.edu.sa (A.S.A.); mssfadel@aucegypt.edu (M.S.); ams06@fayoum.edu.eg (A.M.E.S.)

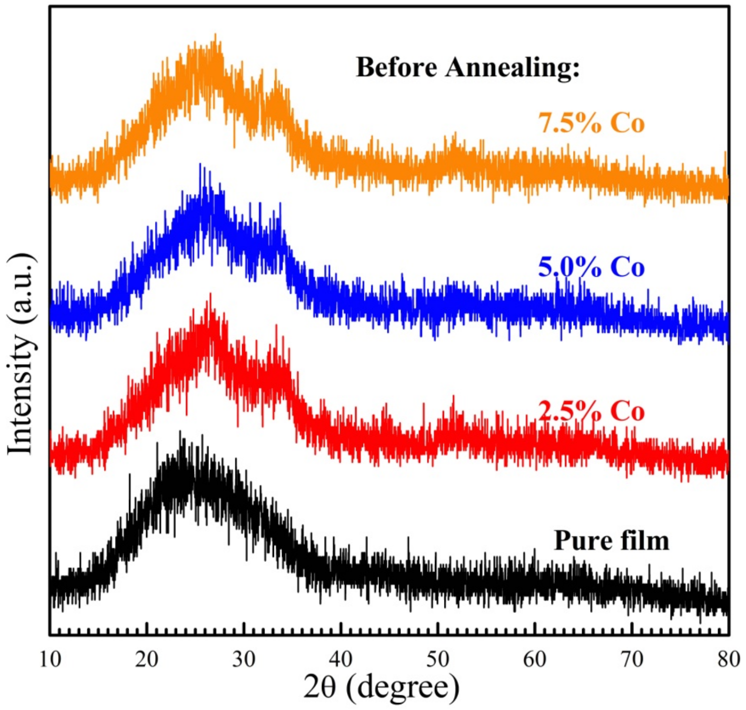

Figure S1. Shows XRD patterns of the as-deposited films (before annealing).

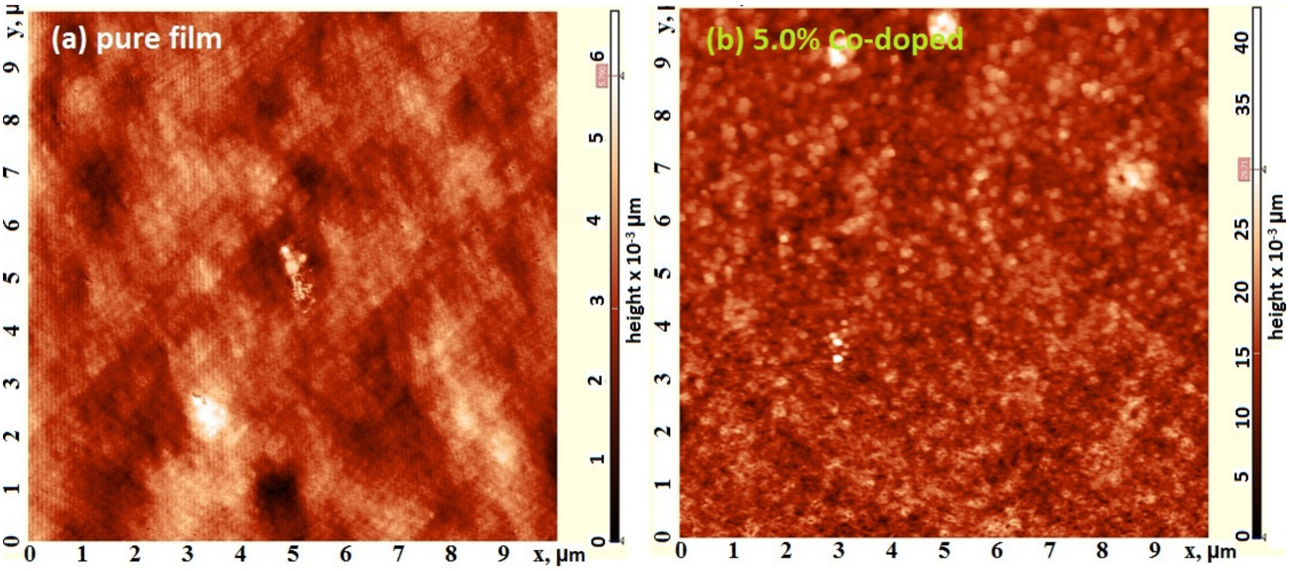

Figure S2. 2D AFM images for (a) pure and (b) 5.0% Co-doped films before annealing.
